# Racial disparities in overall survival among renal cell carcinoma patients with young age and small tumors

**DOI:** 10.1002/cam4.578

**Published:** 2015-12-29

**Authors:** Kendra Schwartz, Julie J. Ruterbusch, Joanne S. Colt, David C. Miller, Wong‐Ho Chow, Mark P. Purdue

**Affiliations:** ^1^ Department of Family Medicine and Public Health SciencesWayne State University School of MedicineDetroitMichigan; ^2^ Population Studies and Disparities Research ProgramKarmanos Cancer InstituteDetroitMichigan; ^3^ Department of OncologyWayne State University School of MedicineDetroitMichigan; ^4^ Division of Cancer Epidemiology and GeneticsNational Cancer InstituteRockvilleMaryland; ^5^ Department of UrologyUniversity of Michigan Medicine SchoolAnn ArborMichigan; ^6^ Department of EpidemiologyDivision of Cancer Prevention and Population SciencesThe University of Texas M.D. Anderson Cancer CenterHoustonTexas

**Keywords:** Age, race, renal cell carcinoma, survival, tumor size

## Abstract

We examined the overall survival of a population‐based cohort of black and white patients with renal cell carcinoma (RCC) to better understand the paradox of poorer RCC survival despite more frequent diagnosis at lower stage among blacks. Renal cell carcinoma patients (699 white, 252 black) diagnosed between 2002 and 2007 in metropolitan Detroit were followed for vital status in the Detroit Surveillance, Epidemiology and End Results (SEER) registry. Hazard ratios (HR) of death for black versus white race and 95% confidence intervals (CIs) were calculated using Cox proportional hazard models stratified by demographic and prognostic factors, and in models successively adjusted for clinical factors, comorbidities, and socioeconomic factors. Mean follow‐up time was 88.4 months for white patients and 89.6 months for black patients (*P* = 0.49), with 202 white deaths and 89 black deaths (*P* = 0.06). While black race was weakly associated with poorer overall survival (*P* = 0.053), black patients <65 years at diagnosis or with tumors <4 cm in size had significantly poorer survival than their white counterparts (HR = 1.46, 95% CI 1.06–2.01 and HR = 2.15, 95% CI 1.51–3.06, respectively). The racial disparities within these two subgroups were minimally affected by adjustment for clinical/treatment factors (HR = 1.49, 95% CI 1.01–2.19 and HR = 1.95, 95% CI 1.27–2.99), but were substantially reduced when renal‐relevant comorbidities were added (HR = 1.30, 95% CI 0.89–1.91 and HR = 1.76, 95% CI 1.16–2.66). After further adjustment for socioeconomic factors, the survival disparities were essentially null (HR = 1.14, 95% CI 0.71–1.85 and HR = 1.15, 95% CI 0.67–1.98). In this population‐based sample of RCC patients, younger black patients and those with small tumors had poorer overall survival than whites. The disparity was explained primarily by racial differences in renal‐relevant comorbidities, particularly chronic renal failure, and socioeconomic deprivation. Future research should focus on younger patients and those with smaller tumors to better understand how these factors may contribute to the survival disparity.

## Introduction

The incidence of renal cell carcinoma (RCC) doubled between 1975 and 2010, from 7.08 to 14.7 per 100,000 1, driven in large part by localized tumors 2, 3, 4, 5, resulting in a stage shift from 43% to 57% Stage 1 tumors between 1993 and 2004 in the National Cancer Database 5. This increase in smaller tumors, especially those ≤4 cm, is thought to be due to increased detection of incidental tumors upon imaging for abdominal complaints 2, 6. Retrospective studies of incidentally discovered tumors indicate that they are indeed smaller and associated with improved survival compared to symptomatic RCC tumors 7, 8, 9.

The increase in RCC incidence is greater among blacks than other racial groups, owing in large part to an increase in localized tumors 3, 10, 11, 12. Despite the fact that black RCC patients are more likely to be diagnosed with early‐stage cancer than their white counterparts, black patients have consistently been reported to have poorer overall survival 3, 10, 13, 14. Investigations of the cause for this survival disparity are rare. A population‐based SEER‐Medicare study of patients 65 years and older observed the disparity to be largely attributable to less use of surgical treatment among blacks compared with whites 13.

Because the SEER‐Medicare study included only elderly patients who were diagnosed between 1986 and 1999, we examined survival patterns among black and white cases of all ages enrolled in the Kidney Cancer Study (KCS), a large case–control study of RCC cases diagnosed between 2002 and 2007. With this resource, we were able to investigate potential influences on overall survival using variables available in SEER, as well as comorbidity and socioeconomic status information from the cases, which is not available in SEER or SEER‐Medicare databases. Additionally, the KCS collected data related to the symptoms at diagnosis, which has been reported by others as being associated with overall survival of RCC patients 15.

## Methods

### Study overview

The KCS is a population‐based case–control study conducted in the metropolitan areas of Detroit, Michigan (Wayne, Oakland, and Macomb Counties) and Chicago, Illinois (Cook County) from 2002 through 2007. Eligible cases were white and black men and women aged 20 to 79 years, with a diagnosis of RCC between 1 February 2002 and 31 July 2007 in Detroit, or 1 January 2003 through 31 December 2003 in Chicago (Hispanic ethnicity was not an inclusion/exclusion criterion). In Detroit, potential cases were identified through the Metropolitan Detroit Cancer Surveillance System, an NCI Surveillance, Epidemiology, and End Results (SEER) program member. This report contains information only for the Detroit cases because SEER data were available to augment clinical information necessary for the analyses and provided vital status information. Cases were followed for vital status through 31 December 2012.

A detailed description of data collection methods was published previously 16. Briefly, participants were recruited by first sending an introductory letter, then by telephone, to participate in a 90‐min epidemiological interview, as well as the optional provision of saliva and blood samples. Participants were offered compensation for their time and effort. At the time of interview, cases were also asked to provide their consent for us to access and review relevant medical records, pathology reports, and tissue samples. If the participant consented to medical record release and tissue samples, a request was sent to the facility and those records/samples were mailed to the study office. The study was approved by the Institutional Review Boards at all participating institutions.

We enrolled 1018 cases from among the 1603 Detroit area patients identified as potentially eligible for this study. This analysis is restricted to Detroit cases that consented to both the interview and medical record review (*N* = 951). The proportions of black and white cases (24%, 66%) are similar to the racial composition of the metropolitan Detroit tri‐county area from which the sample was obtained, which was 25.0% black and 68.9% white in 2000 (U.S. Census) 17.

### Study variables

Demographic and clinical characteristics were obtained from the following sources: (1) participant interview, (2) review of medical records related to the RCC diagnosis, and (3) SEER tumor registry data. From the participant interview, we obtained highest level of educational attainment and detailed medical history regarding comorbidities relevant to renal function, including whether they had ever been told by a doctor that they had diabetes, hypertension, or chronic renal/kidney failure 2 or more years prior to interview. The medical records provided information on presenting signs and symptoms, RCC clinical and pathological characteristics (AJCC stage and Fuhrman grade), and treatment(s). The SEER program provided cancer‐specific information, including tumor size, histology, and vital status.

Using these data, we classified each case into one of three symptom categories at presentation: asymptomatic, local symptoms (e.g., flank or abdominal pain, hematuria), or systemic symptoms (e.g., weight loss, night sweats), similar to previous publications that have demonstrated an association between extent of symptoms at diagnosis and long‐term survival among patients with RCC 6, 7. For analytic purposes, we defined four categories of surgical treatments: (1) no surgery, (2) open radical nephrectomy (ORN), (3) laparoscopic radical nephrectomy (LRN), and (4) nephron‐sparing surgery (NSS; includes both partial nephrectomy and energy‐ablative therapies performed by any approach).

Individual‐level educational attainment was analyzed as less than high school, high school graduate or equivalent, some college, and completion of college and/or a professional degree. A contextual measure of economic deprivation, termed deprivation index (DI), which captures multiple dimensions of the economic and social conditions of neighborhoods including unemployment, poverty, overcrowding, and telephone and automobile availability have been used previously in cancer studies 18, 19, 20. Our DI was created using census‐tract‐level data from the 2000 U.S. Census and the case address at diagnosis. The DI ranges from 0 to 1, where 0 indicates no deprivation (i.e., no unemployment; all households with phone, automobile, and more than one room per person; no individuals living below poverty level) and 1 indicates maximum deprivation. For the purpose of our analysis, DI was categorized into quintiles based on the distribution of deprivation in the metropolitan Detroit area: (Q1 < 0.022, 0.022 < Q2 < 0.035, 0.035 < Q3 < 0.056, 0.056 < Q4 < 0.142, 0.142 < Q5 < 0.531).

### Statistical analyses

We used chi‐square tests to assess racial differences in demographic and cancer‐specific characteristics of the RCC cases, as well as differences in their clinical presentation, diagnosis, and treatment. Overall hazard of death for blacks compared to whites was estimated, with stratification by each demographic and cancer‐related variable. Cox proportional hazards regression models were then stratified by those variables found to be significant in the bivariate model as well as DI (Q1–Q2 vs. Q3–Q5) and a history of renal‐relevant comorbidities (yes/no). The hazard of death associated with black race was evaluated in progressive models adjusted first for the effects of sex, age at diagnosis, tumor size, stage, histology, Fuhrman grade, symptoms at diagnosis, and nephron‐sparing surgery, and then additionally adjusted for history of renal‐relevant comorbidities, and finally adjusted for deprivation index and education. Survival curves were generated using the Kaplan–Meier method to illustrate black–white differences.

All statistical testing was two sided and performed at the 5% significance level (SAS v9.2, SAS Institute, Cary, NC).

## Results

The mean follow‐up time was 88.4 months for white patients (*n* = 699) and 89.6 months for black patients (*n* = 252) (*P* = 0.49). Black patients were more likely than white patients to be of lower AJCC stage (*P* = 0.03), to have RCC of papillary histology (*P* < 0.01), to report either no symptoms or systemic symptoms (*P* = 0.01), to live in a deprived socioeconomic neighborhood (*P* < 0.01), to report fewer years of education (*P* < 0.01), and to report a history of renal‐relevant comorbidities (*P* < 0.01), with the largest difference seen in the report of chronic renal failure (13% vs. 1%; Table 1).

**Table 1:** Comparison of demographic and clinical characteristics of black and white study participants, Detroit Surveillance, Epidemiology and End Results renal cell carcinoma cases diagnosed between 2002 and 2007

	White (*n* = 699)	Black (*n* = 252)	*P* value^a^
	*n* (%)	*n* (%)	
Sex			
Male	396 (0.57)	148 (0.59)	0.57
Female	303 (0.43)	104 (0.41)	
Age at diagnosis			
<65 years	461 (0.66)	182 (0.72)	0.07
≥65 years	238 (0.34)	70 (0.28)	
Mean (SD)	58.8 (11.6)	57.6 (10.5)	0.15
Tumor size (cm)			
≤4 cm	348 (0.50)	135 (0.54)	0.63
4–7 cm	190 (0.27)	66 (0.26)	
>7 cm	147 (0.21)	48 (0.19)	
Unknown	14 (0.02)	3 (0.01)	
Mean (SD)	5.3 (4.9)	4.9 (3.2)	0.12
AJCC stage			
I	452 (0.65)	182 (0.72)	0.03
II	75 (0.11)	29 (0.12)	
III or IV	139 (0.20)	32 (0.13)	
Missing	33 (0.05)	9 (0.04)	
Histology			
Clear cell	533 (0.76)	148 (0.59)	<0.01
Papillary	76 (0.11)	65 (0.26)	
Chromophobe	42 (0.06)	17 (0.07)	
Cystic	36 (0.05)	16 (0.06)	
Other	12 (0.02)	6 (0.02)	
Fuhrman grade			
I	80 (0.11)	21 (0.08)	0.35
II	311 (0.44)	117 (0.46)	
III and IV	204 (0.29)	79 (0.31)	
Missing	104 (0.15)	35 (0.14)	
Surgical treatment			
None	26 (0.04)	10 (0.04)	0.54
Open radical nephrectomy	323 (0.46)	104 (0.41)	
Laparoscopic radical nephrectomy	220 (0.31)	83 (0.33)	
Nephron‐sparing surgery	130 (0.19)	55 (0.22)	
Symptomatology			
Asymptomatic	275 (0.39)	113 (0.45)	0.01
Local	261 (0.37)	71 (0.28)	
Systemic	107 (0.15)	51 (0.20)	
Unknown	56 (0.08)	17 (0.07)	
History of renal‐relevant comorbidities			
None	297 (0.42)	60 (0.24)	<0.01
Hypertension only	288 (0.41)	115 (0.46)	
Diabetes only	26 (0.04)	6 (0.02)	
Hypertension and diabetes (without renal failure)	78 (0.11)	39 (0.15)	
Chronic renal failure	10 (0.01)	32 (0.13)	
Deprivation index^b^			
Q1 (lowest)	204 (0.29)	15 (0.06)	<0.01
Q2	187 (0.27)	21 (0.08)	
Q3	216 (0.31)	15 (0.06)	
Q4	72 (0.10)	98 (0.39)	
Q5 (highest)	20 (0.03)	103 (0.41)	
Education			
Less than high school	81 (0.12)	69 (0.27)	<0.01
High school graduate	262 (0.37)	72 (0.29)	
1–3 years of college	178 (0.25)	78 (0.31)	
College graduate	178 (0.25)	33 (0.13)	
Vital status			
Alive	497 (71.1)	163 (64.7)	0.06
Deceased	202 (28.9)	89 (35.3)	

a *P* value calculations do not include unknown values.

b The deprivation index was developed using data from the 2000 U.S. census and includes the following variables: (1) the proportion of households with no vehicle available; (2) the proportion of households with no telephone available; (3) the proportion of the population 16 years of age and older that is unemployed; (4) the proportion of the population living in a crowded residence; and (5) the proportion of the population living below the poverty level. The first quintile (Q1) indicates a census tract with the lowest economic deprivation; 1.9% of households without a vehicle, 0.4% of households without a telephone, 2.5% unemployment, 0.9% over‐crowding, and 2.1% of the population living below the poverty level. The highest quintile (Q5) has 29.9% of households without a vehicle, 10.4% of households without a telephone, 22.5% unemployment, 9.5% over‐crowding, and 35.1% of the population living below the poverty level.

In Table 2, hazard of death for black race was compared to white race, overall and stratified by the variables in Table 1. Overall, black patients had slightly poorer survival than white patients (HR = 1.28, 95% CI 1.00–1.65). For black patients who were <65 years old at diagnosis (HR = 1.46, 95% CI 1.06, 2.01), had a tumor size of 4 cm or less (HR = 2.15, 95% CI 1.51, 3.06), or were diagnosed at AJCC stage I (HR = 1.67, 95% CI 1.20, 2.33), the survival disparity was substantial. Kaplan–Meier plots and Cox regression models were stratified by tumor size (<4 cm, >4 cm) and age (<65 years, >65 years).

**Table 2:** Univariate hazard of death for black race^a^ stratified by prognostic and demographic variables, Detroit Surveillance, Epidemiology and End Results renal cell carcinoma cases diagnosed between 2002 and 2007

	Black race	*P* value
	HR (95% CI)	
Overall	1.28 (1.00–1.64)	0.053
By sex		
Male	1.18 (0.86–1.62)	0.309
Female	1.43 (0.96–2.14)	0.079
By age at diagnosis		
<65 years	1.46 (1.06–2.01)	0.020
65+ years	1.12 (0.75–1.68)	0.588
By tumor size (cm)		
≤4 cm	2.15 (1.51–3.06)	<0.001
>4 cm	0.81 (0.56–1.19)	0.284
By AJCC stage		
I	1.67 (1.20–2.33)	0.002
II	0.55 (0.23–1.32)	0.178
III	1.73 (0.83–3.60)	0.145
IV	0.95 (0.51–1.78)	0.880
By histology		
Clear cell	1.34 (0.98–1.83)	0.069
Papillary	1.00 (0.54–1.82)	0.987
Chromophobe	1.05 (0.33–3.32)	0.939
Cystic	2.18 (0.84–5.65)	0.110
Other	0.89 (0.27–3.00)	0.856
By Fuhrman grade		
I and II	1.37 (0.94–1.99)	0.104
III and IV	1.30 (0.87–1.93)	0.199
By surgical treatment		
None	1.19 (0.50–2.86)	0.693
Open radical nephrectomy	1.33 (0.94–1.87)	0.108
Laparoscopic radical nephrectomy	1.49 (0.91–2.44)	0.116
Nephron‐sparing surgery	1.13 (0.57–2.24)	0.723
By symptomatology		
Asymptomatic	1.37 (0.92–2.06)	0.126
Local	0.98 (0.61–1.57)	0.915
Systemic	1.09 (0.65–1.83)	0.736
By history of renal‐relevant comorbidities		
None	1.38 (0.83–2.29)	0.212
Hypertension only	1.02 (0.68–1.53)	0.919
Diabetes only	0.78 (0.17–3.59)	0.748
Hypertension and diabetes (without chronic renal failure)	0.85 (0.46–1.58)	0.609
Chronic renal failure	1.25 (0.50–3.13)	0.642
By deprivation index^b^		
Q1 (lowest)	1.52 (0.61–3.82)	0.373
Q2	0.74 (0.30–1.85)	0.524
Q3	0.67 (0.21–2.13)	0.494
Q4	0.94 (0.57–1.57)	0.816
Q5 (highest)	1.46 (0.58–3.68)	0.429
By education		
Less than high school	1.27 (0.75–2.15)	0.378
High school graduate	1.10 (0.70–1.74)	0.673
1–3 years of college	1.41 (0.90–2.22)	0.135
College graduate	0.90 (0.40–2.02)	0.807

a Compared to white race.

b See Table 1 footnote for definition.

**Figure 1:**
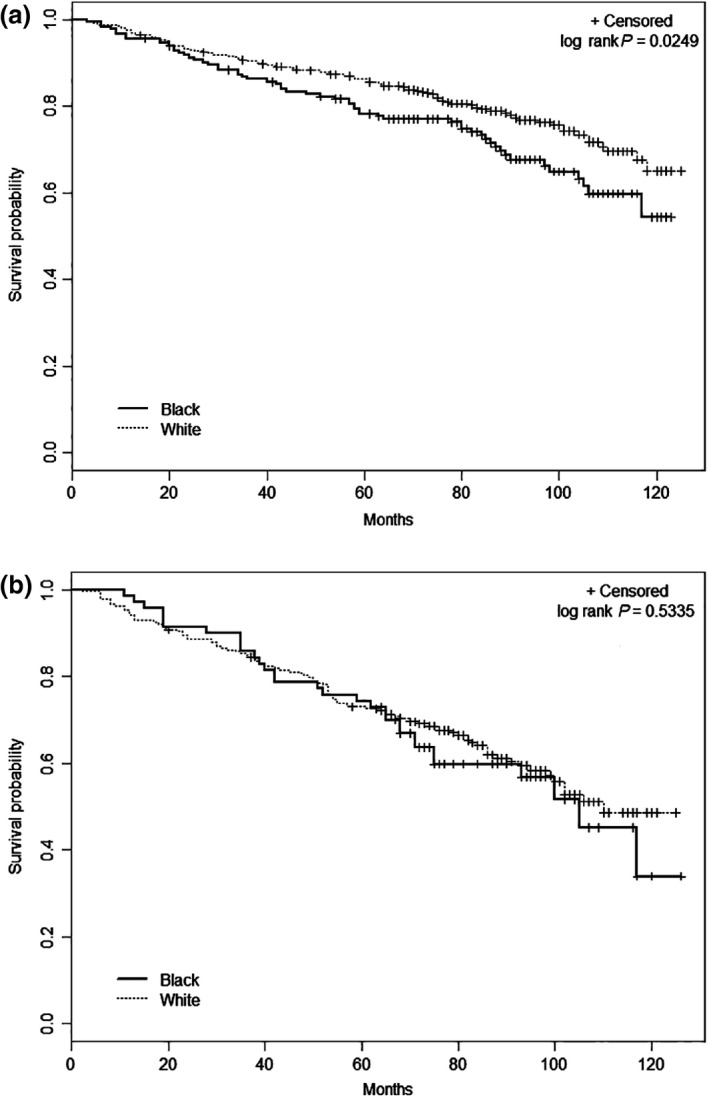
Racial differences in overall survival by age at diagnosis, Detroit Surveillance, Epidemiology and End Results renal cell carcinoma cases diagnosed between 2002 and 2007. (A) Less than 65 years and (B) 65 years or older at diagnosis.

**Figure 2:**
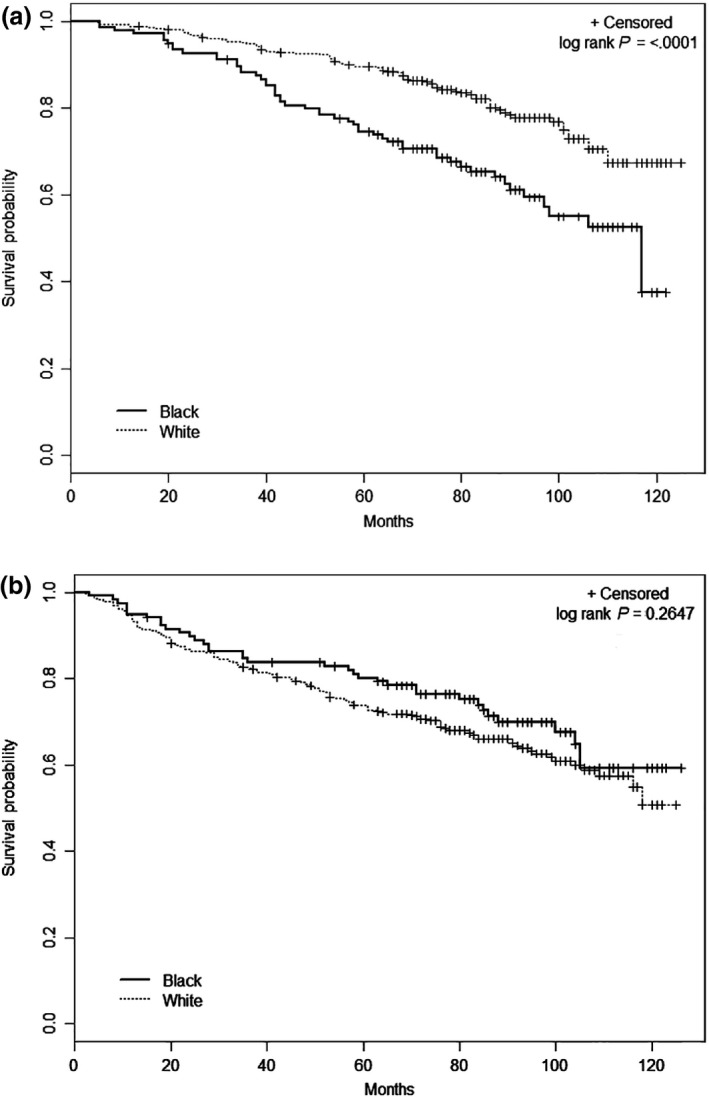
Racial differences in overall survival by tumor size, Detroit Surveillance, Epidemiology and End Results renal cell carcinoma cases diagnosed between 2002 and 2007. (A) Tumors 4 cm or less and (B) tumors >4 cm.

The unadjusted survival disparities for younger age and smaller tumor size are shown in Figures 1 and 2, respectively. There was better survival among young white patients compared to black patients (*P* = 0.025), but survival was similar for the races among patients 65 years and older (*P* = 0.534). White patients with smaller tumors had better overall survival than black patients (*P* < 0.0001), but no significant racial difference was seen for larger tumors (*P* = 0.265).

Table 3 includes the results of successive multivariable modeling for both younger patients and those with smaller tumors. The hazard ratios for black race among patients under 65 years of age (HR = 1.49, 95% CI 1.01–2.19) or with tumors 4 cm or less in size (HR = 1.95, 95% CI 1.27–2.99) were minimally affected by the addition of clinical/treatment factors into the models. However, the HRs were weakened when comorbidities were added as covariates (HR = 1.30, 95% CI 0.89–1.91 and HR = 1.76, 95% CI 1.16–2.66, respectively), and approached the null with additional adjustment for socioeconomic factors (HR = 1.14, 95% CI 0.71–1.85 and HR = 1.15, 95% CI 0.67–1.98, respectively). The pattern for low AJCC stage (I and II) is similar to that seen for smaller tumors, as would be expected because tumor size is a component of the RCC staging criteria. The strata of deprivation index and renal‐relevant comorbidities showed insignificant HRs throughout the sequential models.

**Table 3:** Relative hazard of death for black race compared to white race, sequentially adjusted for prognostic variables, Detroit Surveillance, Epidemiology and End Results renal cell carcinoma cases diagnosed between 2002 and 2007

	Unadjusted	Model 1	Model 2	Model 3
		Clinical/treatment	Plus comorbidities	Plus SES
	HR (95% CI)	HR (95% CI)	HR (95% CI)	HR (95% CI)
Overall	1.28 (1.00–1.64)	1.32 (0.98–1.79)	1.17 (0.87–1.57)	0.93 (0.65–1.35)
Age at diagnosis^a^				
<65	1.46 (1.06–2.01)	1.49 (1.01–2.19)	1.30 (0.89–1.91)	1.14 (0.71–1.85)
≥65	1.12 (0.75–1.68)	1.04 (0.64–1.68)	0.86 (0.53–1.38)	0.64 (0.36–1.14)
Tumor size^a^				
≤4 cm	2.15 (1.51–3.06)	1.95 (1.27–2.99)	1.76 (1.16–2.66)	1.15 (0.67–1.98)
>4 cm	0.81 (0.56–1.19)	0.93 (0.59–1.47)	0.80 (0.51–1.26)	0.74 (0.44–1.27)
AJCC stage^a^				
I	1.67 (1.20–2.33)	1.67 (1.17–2.40)	1.33 (0.90–1.94)	0.91 (0.56–1.47)
II–IV	1.10 (0.73–1.67)	1.00 (0.61–1.62)	0.95 (0.58–1.54)	0.88 (0.49–1.60)

Model 1: Adjusted for sex, age at diagnosis, tumor size, AJCC stage, histology, Fuhrman grade, symptomatology, nephron‐sparing surgery.

Model 2: Adjusted for all model 1 variables plus history of diabetes, hypertension, and chronic renal failure.

Model 3: Adjusted for all model 2 variables plus education level and deprivation index.

a For stratified analyses, models are not adjusted for that strata variable.

We tested the proportional hazards assumption in the multivariate models by adding an interaction term with race and follow‐up time to the final models. This interaction term was not significant for the overall model or for the stratified models.

The distribution of the DI and renal‐relevant comorbidities by race was evaluated to determine if the pattern differed for the age and tumor size subgroups. The racial distributions within the age and tumor size groups were very similar; black patients were more likely to live in areas of high economic deprivation and reported a higher number of renal‐relevant comorbidities in the same proportion regardless of age or tumor size (data not shown).

To be confident that nonsurgically treated patients (*N* = 36) were not influencing the results, we repeated the multivariable analyses after excluding them from the models and the results were nearly identical to those provided in Tables 2 and 3 (data not shown).

## Discussion

In this population‐based sample of RCC patients, we found that overall survival for all black patients was slightly poorer than their white counterparts. However, black patients <65 years old at diagnosis and those presenting with small tumors had significantly poorer overall survival than white patients. The racial disparity in survival within these two subgroups was no longer apparent after model adjustment for renal‐relevant comorbidities and socioeconomic indicators. However, adjusting for presenting symptoms and treatment type had no effect on survival.

Our results suggest that renal‐relevant comorbidities (hypertension, diabetes, and chronic renal failure) are major contributors to the poorer overall survival among young black patients and black patients with smaller tumors. Moreover, fewer blacks reported having no renal‐relevant comorbidities, and chronic renal failure was reported significantly more often by black patients (13% vs. 1%). Berndt et al. 13, using the SEER‐Medicare database, also looked at black/white RCC survival disparities among patients 65 years and older, a group for which we found no statistically significant survival disparity. They found that lack of nephrectomy accounted for much of the poorer survival among black Medicare patients. Furthermore, among patients undergoing nephrectomy, blacks had worse survival; however, adjustment for end‐stage renal disease reduced the racial disparity. Hypertension‐related end‐stage renal disease is more common and typically more severe among blacks compared to whites 21, and appears to be mainly explained by the presence of *APOL1* genetic variants that are strongly associated with risk of selected nonmalignant kidney diseases in populations of African ancestry 22. Additionally, chronic renal failure appears to be a stronger risk factor for RCC among blacks than whites, possibly due to pathologic changes related to loss of renal function 23, 24. These same pathological changes also may lead to poorer survival, particularly among black RCC patients.

Our finding that socioeconomic factors, both neighborhood deprivation and individual education level, was important in attenuating the racial survival disparity among younger patients and in patients with smaller tumors has not been previously reported. The black patients in our population were of significantly lower socioeconomic status than the white patients, with over 80% of blacks living in the most deprived neighborhoods, regardless of their age or tumor size. It has been shown that socioeconomic status differentially influences patterns of morbidity and mortality by race, with black patients more likely to be negatively affected than whites with similar socioeconomic profiles 25, 26. Additionally, racial residential segregation is thought to represent a fundamental cause of racial disparities in health 27, and metropolitan Detroit is often cited as one of the most racially segregated urban areas in the United States 28. Lower socioeconomic status can result in lower access to care, which can affect survival. Although both black and white patients in our study had similar rates of surgery and nephron‐sparing procedures, there may be nonmeasured access factors, such as treatment delays or surgical complications, which may be affecting survival. Other aspects of low socioeconomic status, such as stress, lack of social support systems, and environmental exposures, could not be evaluated in this study and should be the focus of future investigations of racial disparities in RCC survival.

Chow et al. 14, using national SEER data, reported that black RCC patients had poorer 5‐year relative survival than whites regardless of age, sex, tumor stage or size, histological subtype, or surgical treatment. However, information on comorbidities and socioeconomic deprivation are not available within the SEER database, unlike our study. In contrast, we found a survival disparity between black and white patients, particularly among younger patients and those with smaller tumors, and these racial disparities were largely explained by more prevalent comorbidities and social deprivation among blacks. Our observations underscore the importance of socioeconomic contextual environment in influencing RCC survival. Yet it is unclear why younger black patients and those with smaller tumors appear to be more affected by deprivation compared to whites than older black patients and those with larger tumors. One important factor related to age may be the availability of Medicare insurance at age 65 years and older.

Petard et al. 15 demonstrated that symptomatology (categorized as asymptomatic, renal specific symptoms and systemic symptoms) was predictive of overall survival independent of AJCC stage and Fuhrman grade, and was a better predictor than ECOG functional status. Within our population, black patients had a higher prevalence of systemic symptoms, yet adjusting for symptomatology did little to attenuate the elevated hazard ratios seen.

This population‐based study provided a unique opportunity to investigate racial differences in RCC survival among both young and elderly patients, with adjustment for both prognostic and socioeconomic factors. However, several limitations must be acknowledged. The renal‐relevant comorbidities used in this study were based on self‐report and were not verified. Although it is likely that most patients know their diagnosis, especially of more common diseases like hypertension and diabetes, it is unclear how knowledgeable they would be of chronic renal failure unless they were receiving dialysis. In fact, approximately 70% of those reporting chronic renal failure also reported dialysis, regardless of tumor size. The study sample size also may be considered low, especially for black cases, resulting in wider confidence intervals as well as the possibility of false positive findings. Finally, we were able to interview only those RCC cases that had not died prior to contact. The most aggressive types of RCC are thus underrepresented in this sample.

In conclusion, our findings suggest that in metropolitan Detroit, the poorer survival experienced by black RCC patients compared to white patients, despite being diagnosed at a more favorable stage of disease, is mainly attributable to racial differences in comorbidities, particularly chronic renal failure, and factors associated with low socioeconomic status. These factors particularly affect younger black patients and those with smaller tumors; however, the reasons for this remain unclear. The associations are likely to be interrelated and complex and deserve further research.

## Conflicts of Interest

None declared.
